# Identification and Functional Characterization of *ZmSCYL2* Involved in Phytosterol Accumulation in Plants

**DOI:** 10.3390/ijms241210411

**Published:** 2023-06-20

**Authors:** Chenchen Zhang, Wanlu Ma, Minyan Xu, Tao Li, Guomin Han, Longjiang Gu, Meng Chen, Mengting Zhang, Beijiu Cheng, Xin Zhang

**Affiliations:** National Engineering Laboratory of Crop Stress Resistance Breeding, School of Life Sciences, Anhui Agricultural University, Hefei 230036, China

**Keywords:** phytosterols, GWAS, *ZmSCYL2*, transgenic, *Arabidopsis*, tobacco

## Abstract

Phytosterols are natural active substances widely found in plants and play an important role in hypolipidemia, antioxidants, antitumor, immunomodulation, plant growth, and development. In this study, phytosterols were extracted and identified from the seed embryos of 244 maize inbred lines. Based on this, a genome-wide association study (GWAS) was used to predict the possible candidate genes responsible for phytosterol content; 9 SNPs and 32 candidate genes were detected, and *ZmSCYL2* was identified to be associated with phytosterol accumulation. We initially confirmed its functions in transgenic *Arabidopsis* and found that mutation of *ZmSCYL2* resulted in slow plant growth and a significant reduction in sterol content, while overexpression of *ZmSCYL2* accelerated plant growth and significantly increased sterol content. These results were further confirmed in transgenic tobacco and suggest that *ZmSCYL2* was closely related to plant growth; overexpression of *ZmSCYL2* not only facilitated plant growth and development but also promoted the accumulation of phytosterols.

## 1. Introduction

Sterols are triterpenoids with steroid skeleton structures and are important components of phospholipid bilayer membranes [1], whose functions are related to membrane fluidity and permeability as well as embryogenesis [2,3]. Sterols are present in the tissues of various organisms and can be classified into three major groups according to their origin. Sterols of plant origin are usually referred to as phytosterols, those of animal origin as zoosterols, and those produced by yeasts and fungi as ergosterols [4]. Phytosterols regulate a number of physiological activities in plant growth and development, such as cell division, plant defense, polarity, and morphogenesis [5,6,7].

Phytosterols have been shown to reduce plasma total and low-density lipoprotein (LDL) cholesterol levels in human subjects, thereby preventing hypercholesterolemia and cardiovascular disease, as well as having anti-cancer, anti-inflammatory and antioxidant properties [8,9,10,11]. Interest in phytosterols is growing due to their potential benefits for human health. Mammals (including humans) are unable to produce phytosterols and, therefore, must rely on dietary sources, mainly plant-based foods [12]. The content of phytosterols varies depending on the food sources, being higher in vegetable oils, nuts, grains, and legumes but lower in vegetables [13]. Phytosterols have become one of the popular functional nutritional factors in the international food market. Engineering solutions for the biofortification of food crops by enhancing the natural phytosterol content have attracted concern.

The accumulation of phytosterols is influenced by both synthetic and transport processes. The sterol biosynthesis process has been studied in detail [14,15] and is mainly synthesized via the mevalonate pathway of isoprenoid biosynthesis using acetyl-CoA as a precursor in a multi-step reaction catalyzed by various enzymes [16]. Various rate-limiting enzymes regulate and influence sterol synthesis during this process, and most of them have been cloned, and their functions confirmed by in vitro assays or complementary analysis. The site of phytosterol biosynthesis occurs in the endoplasmic reticulum but does not accumulate at this site; they appear to be rapidly transported via the Golgi apparatus to the plasma membrane and accumulate mainly there [17]. However, the molecular mechanisms of sterol transport are less reported, and the available studies suggest two main modes of intracellular sterol transport, one involving transport proteins (an ATP-binding cassette and sterol carrier proteins) and the other trafficking through vesicles [18,19,20]. How sterols are transported between different organelles and cells is still not fully understood and is a subject for further research.

Vesicle trafficking plays an important role in delivering a diverse range of cargoes between different membrane systems in eukaryotes [21]. In general, the transport initiates from vesicle budding from a donor compartment and subsequently targets and fuses with an acceptor compartment [22]. Some proteins, such as coat proteins, are required to regulate vesicle trafficking during this process [23,24]. Three types of vesicles can be classified according to their surface-coated proteins: coat protein complex I (COPI), coat protein complex II (COPII), and clathrin-coated vesicles (CCVs) [25,26], in which CCVs were first discovered to mediate protein trafficking from the Golgi to the plasma membrane. Proteins included in the category of CCVs include three clathrin heavy chains (CHCs), three clathrin light chains (CLCs), and various adaptor protein complexes [27].

SCYL2 (SCY1-LIKE2) belongs to the SCY1-like family [28] and was identified through subcellular proteomics as a component of CCVs that regulates clathrin-mediated endocytosis by phosphorylating plasma membrane adaptor AP2. Duwel et al. confirmed the interaction between SCYL2 and components of the clathrin machinery by co-immunoprecipitation but failed to detect any related kinase activity; instead, they found that the N-terminal kinase domain of SCYL2 mediated the endocytic membrane interactions, while part of the C-terminal structure can interact with clathrin-AP1 complex components [29]. Conner et al. found that SCYL2 was involved in the transport of CCVs between the Golgi and endosomal system in animals and played an important role in the development of animal cells [30]. SCYL2 was also indispensable for neuronal function and survival in the animal brain [31,32,33]. Jung et al. found that SCYL2A and SCYL2B in *Arabidopsis* were essential for plant cell growth and root hair development. SCYL2 played vital roles in plant cell developmental processes via clathrin-mediated vesicle membrane trafficking [34]. *OsSCYL2* was reported to regulate plant innate immunity in rice [35]. However, the function of *ZmSCYL2* has not been reported.

In this study, we identified the candidate gene *ZmSCYL2* (GRMZM2G061681) that is responsible for phytosterol content and verified its function in transgenic *Arabidopsis* and tobacco and found that *ZmSCYL2* promoted plant growth and phytosterol accumulation.

## 2. Results

### 2.1. Variation in Phytosterol Content among the Maize Inbred Lines

In this study, three major phytosterols in maize (*Zea mays*) embryos stripped from maize seeds were extracted and identified as campesterol, stigmasterol, and β-sitosterol based on the GC retention times and mass spectrometry data of the isolates. The 3 major sterols and the total sterol content in embryos of 244 maize inbred lines were quantified by GC-MS. The data are presented in Supplementary Table S1 and used for genome-wide association study (GWAS) analysis. Among all maize lines, β-sitosterol was the most abundant phytosterol, with a range of 0.548–11.649 mg/g, representing approximately 61.81–91.54% of the total sterol content. The contents of campesterol and stigmasterol were relatively lower, ranging from 0.052–2.888 mg/g of campesterol and 0.046–3.107 mg/g of stigmasterol, accounting for 4.62–28.45% and 1.34–25.81% of the total phytosterol content, respectively. The phytosterol content varied significantly between maize varieties, with some of them having a total sterol content as high as 13.722 mg/g, while others were as low as 0.696 mg/g. In addition, the proportion of phytosterols composition varied depending on the maize variety. Varietal differences resulting in differences in sterol contents and composition ratios provided a basis for the purposeful screening of maize varieties with high sterol contents.

### 2.2. Gene Cloning and Bioinformatics Analysis of ZmSCYL2

**Figure 1:**
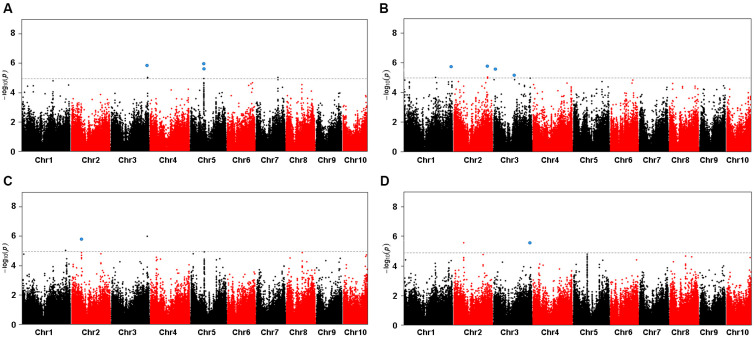
Manhattan plot of a mixed linear model (MLM) for phytosterol contents analysis. The dashed horizontal line depicts the Bonferroni-adjusted significance threshold (*p* = 1.04 × 10^−5^). Nine significant SNPs that met this level are enlarged and marked with blue dots, (**A**) campesterol contents, (**B**) stigmasterol contents, (**C**) β-sitosterol contents, and (**D**) total sterol contents.

The maize SNP50 BeadChip used in this study contained a total of 558,529 SNPs. GWAS was performed using a mixed linear model (MLM), and both kinship relationship (K matrix) and population structure (Q matrix) were taken into account to avoid spurious associations [36]. As shown in Figure 1, a total of 9 SNPs significantly associated with sterol content were detected on Chr 1, 2, 3, 5, and 8. Gene models containing or adjacent to the SNPs above the Bonferroni significance threshold were selected as candidate genes. Thirty-two candidate genes directly associated with SNPs were found using the B73 Maize Gene Database (http://www.maizegdb.org/ (accessed on 8 September 2021)), including 21 genes with annotated protein-encoding functions and 11 genes with uncharacterized proteins. Among these genes, we identified a gene with high protein sequence homology to *Arabidopsis* SCYL2, which contains a clathrin-binding motif (SLLDLL) and a conserved protein kinase domain. Therefore, we designated it *ZmSCYL2*.

### 2.3. Gene Cloning and Bioinformatics Analysis of ZmSCYL2

**Figure 2:**
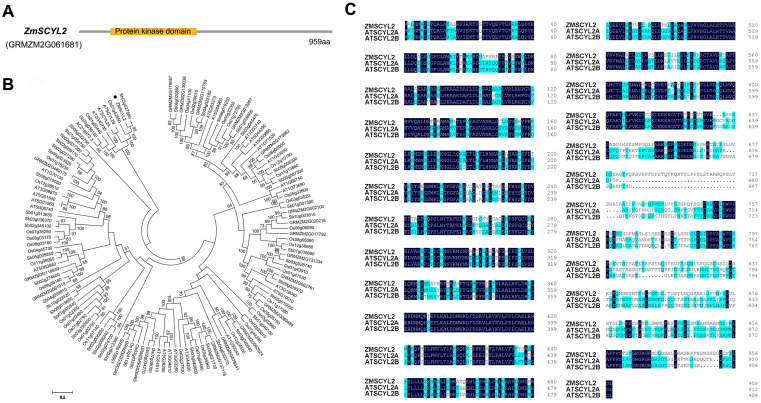
Schematic characterization of ZmSCYL2 and multiple alignments with other homologs. (**A**) Conserved domains analysis of ZmSCYL2. There is a conserved protein kinase domain in ZmSCYL2, localized at ~39–344 aa. (**B**) Phylogenetic analysis of ZmSCYL2 homologs by use of Neighbor-Joining analysis. The numbers above the nodes are percentages of bootstrap confidence levels from 10,000 replicates. Branch length represents the number of amino acid changes per site in accordance with the scale bar. Studied gene is indicated by a black circle. All sequence data can be found in GenBank. (**C**) Multiple sequence alignment with Arabidopsis homologous sequences. Identical and similar residues are shaded in dark blue and light blue, respectively.

The full-length sequence of *ZmSCYL2* was isolated from maize inbred lines by RT-PCR using gene-specific primers. The sequence was 2880 bp in length and encoded a protein of 959 amino acids with a predicted molecular mass of 103.04 kDa and a calculated pI of 6.74. Protein structure analysis revealed that ZmSCYL2 had a conserved protein kinase domain (Figure 2A) at the N-terminal and a clathrin-binding motif at the C-terminal, which were also found in other SCYL-type proteins in various species.

Phylogenetic analysis was constructed to assess the relationship between maize and other species. As shown in Figure 2B, ZmSCYL2 had significant homology with AtSCYL2A and AtSCYL2B of *Arabidopsis*. In addition, the protein sequences of ZmSCYL2, AtSCYL2A, and AtSCYL2B were subjected to multiple sequence alignment. The results indicated that the amino acid sequence of ZmSCYL2 had high homology with AtSCYL2A and AtSCYL2B (68.87% and 69.88%, respectively), and all of them have a conserved protein kinase domain (Figure 2C).

**Figure 3:**
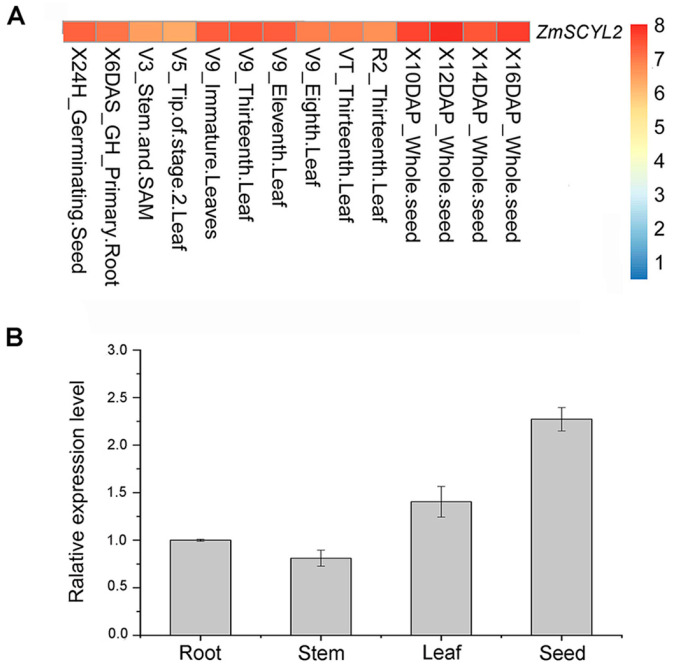
Expression profiles of *ZmSCYL2*. (**A**) A heatmap illustrating levels of the gene expression of *ZmSCYL2* in different tissues from various developmental stages. The expression data from the maize gene expression atlas are available at MaizeGDB for different tissues at specific developmental stages. Normalized gene expression values are shown in different colors that represent the levels. Blocks with red colors indicate high expression levels, whereas blue blocks indicate lower expression levels. (**B**) Relative expression levels of *ZmSCYL2* by qRT-PCR in different tissues (root, stem, leaf, and seed) in maize. The expression level in the root was defined as 1.0. Data represent the mean ± SD of three biological replicates.

To gain insight into the biological functions of *ZmSCYL2*, we obtained the gene expression data from the maize gene expression atlas available at MaizeGDB of different tissues at specific developmental stages and constructed a heatmap to investigate the expression profiles. As shown in Figure 3A, although *ZmSCYL2* was expressed in all tissues, the expression levels varied in different tissues, with *ZmSCYL2* being expressed mainly in leaves and seeds. The specific expression pattern of *ZmSCYL2* in four tissues of maize B73 inbred lines (root, stem, leaf, and seed) was analyzed by qRT-PCR. The results showed that the *ZmSCYL2* transcript was predominantly expressed in seeds, followed by leaves, and weakly in roots and stems (Figure 3B).

**Figure 4:**
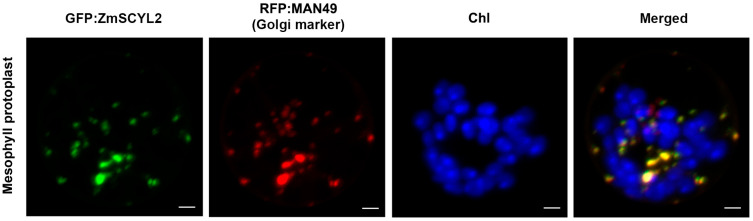
Subcellular localization of ZmSCYL2-GFP fusion proteins in mesophyll protoplast of maize. Laser-scanning confocal microscopy images show fluorescence (GFP) and merged images. ZmSCYL2-GFP signal is indicated in green, MAN49, used as a Golgi maker, is indicated in red and chlorophyll autofluorescence (Chl) is in blue. The overlap of green and red produce yellow fluorescence. Bars = 5 μm.

To investigate the subcellular localization of ZmSCYL2, a pCAMBIA1305-35S-ZmSCYL2-GFP recombinant vector was constructed by inserting the coding sequence without the termination codon of *ZmSCYL2* in frame with the GFP reporter driven by CaMV35S promoter, and transiently expressed the recombinant vector in maize protoplast cells. Using RFP: MAN49 as a fluorescent protein marker for Golgi, the GFP signal of ZmSCYL2 was found to overlap with the RFP fluorescent signal of Golgi, indicating that ZmSCYL2 functions in Golgi (Figure 4).

### 2.4. Expression of ZmSCYL2 Affects Phytosterol Content in Arabidopsis

**Figure 5:**
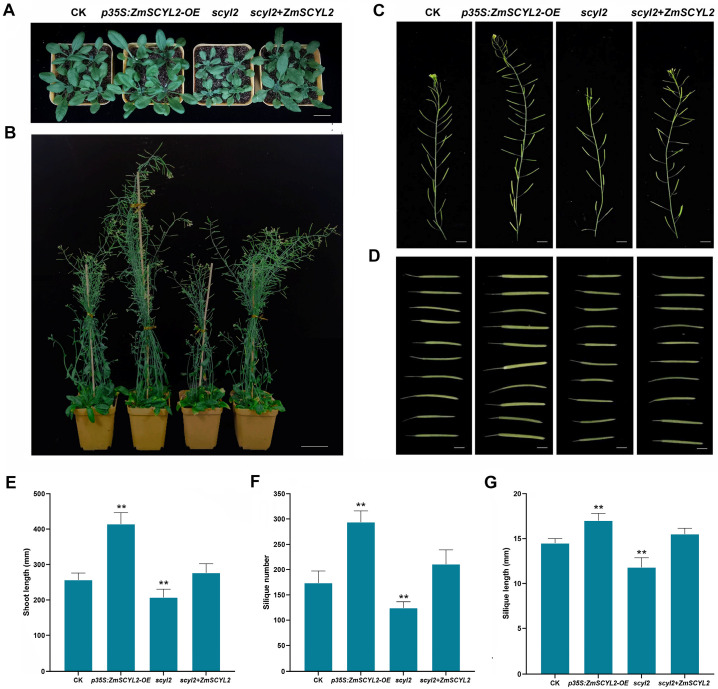
*ZmSCYL2* is important in plant growth and development in *Arabidopsis*. (**A**) Phenotypic comparison between CK (wild-type Col-0), *ZmSCYL2-OE*, *scyl2* and *scyl2* +* ZmSCYL2* at 10 days seedlings. Bars = 2 cm. (**B**) Phenotypic comparison between mature CK and transgenic lines. Bars = 5 cm. (**C**) Comparison of shoot length between CK, *ZmSCYL2-OE*, *scyl2*, and *scyl2* +* ZmSCYL2*. Bars = 1 cm. (**D**) Comparison of silique length. Bars = 4 mm. (**E**) Average shoot length of CK, *ZmSCYL2-OE*, *scyl2*, and *scyl2* +* ZmSCYL2*. (**F**) Average silique number of CK, *ZmSCYL2-OE*, *scyl2*, and *scyl2* +* ZmSCYL2*. (**G**) Average silique length of CK, *ZmSCYL2-OE*, *scyl2*, and *scyl2* +* ZmSCYL2*. Values are means ± SD from 40 plants. Asterisks indicate significant differences between control and transgenic lines (** means *p* < 0.01, by *t*-test, compared with control).

The phenotypes of control (CK, *Arabidopsis* wild-type Col-0) and transgenic plants (overexpressed, mutant, and complemented mutant *Arabidopsis*) were observed, as shown in Figure 5A–D.

Compared to the control group, the phenotype of the overexpression line (*ZmSCYL2-OE*) changed significantly after 10 days of germination with faster development, larger leaves, and longer shoots, whereas mutant plants (*scyl2*) grew more slowly and had shorter shoots (Figure 5A). When the plants were grown to 45 days to full maturity, more pronounced differences in plant height, siliques number, and length could be observed (Figure 5B). The average height of the control, *ZmSCYL2*-*OE*, *scyl2*, and complemented mutant plants (*scyl2* +* ZmSCYL2*) were 256.4, 414.1, 207.2, and 276.4 mm, respectively (Figure 5C), the average number of siliques was 173.4, 293.8, 124.2, and 210.6 cm, respectively (Figure 5D), and the average length of siliques was 14.5, 17.0, 11.8, and 15.5 mm, respectively (Figure 5E–G). These data suggested that the phenotypes of *Arabidopsis* were closely related to the expression levels of *ZmSCYL2*, with better plant growth when *ZmSCYL2* was overexpressed, and conversely, the lack of this gene led to slow plant growth.

**Figure 6:**
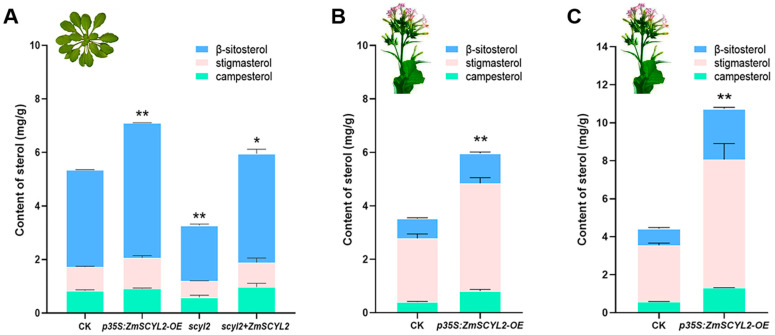
Analysis of sterol content in CK and transgenic plants. (**A**) Phytosterol contents of *Arabidopsis* seeds in CK, *ZmSCYL2-OE*, *scyl2*, and *scyl2* +* ZmSCYL2*. (**B**) Phytosterol contents of tobacco leaves in CK and *ZmSCYL2-OE*. (**C**) Phytosterol contents of tobacco seeds in CK and *ZmSCYL2-OE*. Data represent the mean ± SD of three biological replicates. Asterisks indicate significant differences between control and transgenic lines (* means *p* < 0.05, ** means *p* < 0.01, by *t*-test, compared with control).

In this study, phytosterols were extracted and quantitatively analyzed from the seeds of control, *ZmSCYL2-OE*, *scyl2*, and *scyl2* + *ZmSCYL2* plants (Figure 6A). It was found that the major sterols were β-sitosterol, campesterol, and stigmasterol in *Arabidopsis* seeds. The total sterol content in *ZmSCYL2-OE* was about 7.11 mg/g, in which campesterol, stigmasterol, and β-sitosterol were 0.91 mg/g, 1.14 mg/g, and 5.04 mg/g, respectively. The total content of sterol in the control group was about 5.34 mg/g (campesterol 0.83 mg/g, stigmasterol 0.88 mg/g, and β-sitosterol 3.63 mg/g). The total content of sterols in *scyl2* was about 3.27 mg/g (campesterol 0.58 mg/g, stigmasterol 0.62 mg/g, and β-sitosterol 2.07 mg/g). In *scyl2* + *ZmSCYL2*, the total sterol was increased to 5.95 mg/g (campesterol 0.97 mg/g, stigmasterol 0.92 mg/g, and β-sitosterol 4.06 mg/g). It could be seen that the sterol content was highest in the overexpression line, followed by the control, and lowest in the mutant, and the sterol content of the complemented mutant plant was comparable to that of the control, indicating that sterol accumulation was positively correlated with gene expression, and the expression level of *ZmSCYL2* affected sterol accumulation.

### 2.5. Effects of ZmSCYL2 on Growth and Phytosterol Content in Tobacco

**Figure 7:**
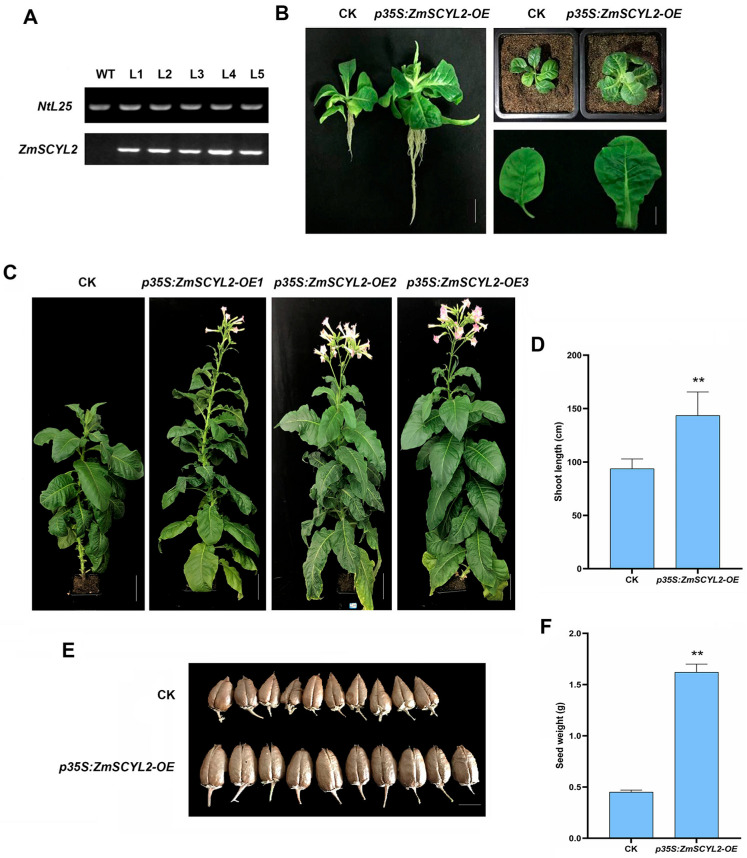
Overexpression of *ZmSCYL2* promotes growth and development in tobacco. (**A**) Relative expression levels of *ZmSCYL2* were measured by qRT-PCR in CK and *ZmSCYL2-OE* lines. *NtL25* served as a loading control. (**B**) Phenotypic comparison between CK and *ZmSCYL2-OE* one week after transfer from the medium to the pots. Bars = 3 cm. (**C**) Phenotypic comparison between mature CK and *ZmSCYL2-OE* lines. Bars = 15 cm. (**D**) Comparison of shoot length. (**E**) Comparison of pods between CK and *ZmSCYL2-OE*. Bars = 1 cm. (**F**) Comparison of seed weight per 10 pods. Values are means ± SD from 20 plants. Asterisks indicate significant differences between control and transgenic lines (** means *p* < 0.01, by *t*-test, compared with control).

Transgenic plants of tobacco were obtained by the leaf disc method using the same *Agrobacterium* strain containing the *ZmSCYL2* recombinant plasmids as *Arabidopsis* transformation. Positive plants (*ZmSCYL2-OE*) were identified by qRT-PCR (Figure S1), and the negative plants served as controls (Figure 7A). In observing the growth of the plants, it was found that, compared to the control, the overexpression seedlings developed faster after 1 week of growth following transfer from the medium to the pots (Figure 7B). When the tobacco plants grew to maturity, flowering and fruiting in *ZmSCYL2-OE* lines were also 1–2 weeks earlier than in the control, and the difference in plant height between the two became more obvious, with the *ZmSCYL2-OE* lines reaching an average plant height of 143.52 cm compared to 93.75 cm for the control, about 2/3 of the *ZmSCYL2-OE* plants (Figure 7C,D). The seeds of the *ZmSCYL2*-*OE* lines were larger and full, weighing about 1.62 g per 10 pods, while the seeds of the control were relatively dry and had a lower fruit set rate, weighing only 0.45 g per 10 pods (Figure 7E,F).

The phytosterols in tobacco were mainly composed of campesterol, stigmasterol, and β-sitosterol. Different from β-sitosterol which was the main phytosterol in *Arabidopsis* and maize seeds, the most abundant sterol in tobacco was stigmasterol accounting for about 50–70% of the total sterol, followed by β-sitosterol for about 10–30% and campesterol for about 5–20%. The sterol content in tobacco seeds was higher than that in leaves. The total sterol content of *ZmSCYL2*-*OE* seeds was 2-fold higher than that of the control (Figure 6C), and the sterol content of *ZmSCYL2*-*OE* leaves was 1.25-fold higher than that of the control (Figure 6B).

## 3. Discussion

Phytosterols have a reputation as “the key of life” and have important physiological and nutritional value to both plants and the human body. Improving sterol accumulation in cereals by applying metabolic engineering is regarded as an important strategy. There are many kinds of sterols in maize, among which campesterol, stigmasterol, and β-sitosterol are dominant. The composition of sterols in maize varied with different varieties. Generally, β-sitosterol accounted for the highest proportion, exceeding 60% of total sterols, and even could reach 90%.

A genome-wide association study (GWAS), which is based on genetic linkage disequilibrium (LD) in a panel including a large number of genotypes representing broadly natural variations, has been used as an approach for exploring the molecular basis and identifying SNPs of complex quantitative traits [37]. GWAS provides a higher resolution mapping of genetic loci associated with agronomic traits compared to transcriptome data analysis. By utilizing a dense panel of genetic markers, GWAS can pinpoint specific regions of the genome that contribute to the observed variations in agronomic traits. By leveraging the advantages of GWAS, we can identify candidate genes and genetic markers associated with agronomic traits, which can inform breeding programs and contribute to the development of improved maize varieties. In this study, GWAS were used to identify phytosterol content and predict the possible candidate genes responsible for phytosterol accumulation. According to the results of GWAS, 9 SNPs and 32 candidate genes related to phytosterol content were identified with high confidence, and *ZmSCYL2* related to phytosterol content was preliminarily identified.

*ZmSCYL2* belongs to the SCY1-like gene family, which is considered to be a relatively conservative protein in evolution, with a protein kinase domain at the N-terminal and one or more spiral curl regions at the C-terminal [28]. The expression pattern of *ZmSCYL2* in different tissues of maize was investigated, and it was found that *ZmSCYL2* was expressed in roots, stems, leaves, and seeds with high expression, among which the expression level of *ZmSCYL2* in seeds was the highest, about twice as much as that in the others (Figure 3B). The expression analysis results revealed that *ZmSCYL2* plays an important role in the entire process of maize growth and development, especially in the development of seeds.

Subcellular localization showed that ZmSCYL2 was located in the Golgi apparatus (Figure 4), suggesting that its function may be closely related to the activity of the Golgi apparatus. The functions of the Golgi apparatus are numerous, involving protein glycosylation, protein hydrolysis, membrane transformation, and participation in the secretory activities of cells. Conner et al. first identified in animals that SCYL2 was involved in the substance transport between the Golgi apparatus and the endosomal system by clathrin-coated vesicles [30]. Borner et al. also found that SCYL2 plays a role in regulating clathrin function in the trans-Golgi network and compartments [31]. The SCYL2 protein in *Arabidopsis* has also been shown to be involved in the transport of clathrin-coated vesicles. It is speculated that the ZmSCYL2 protein in maize is also involved in the transport of sterols through clathrin-coated vesicles, and the overexpression of *ZmSCYL2* may be conducive to the accumulation of sterols in maize.

Jung et al. cloned two homologous genes (*AtSCYL2A* and *AtSCYL2B*) from *Arabidopsis*; they found *SCYL2A* and *SCYL2B* are essential for plant growth. Mutation of both *SCYL2A* and *SCYL2B* genes led to severe dwarfism, while seed development was also affected [34]. Yao et al. found that the mutant plant (*scyl2*) was shorter and more susceptible to disease than WT at the tillering stage of rice. The grain size of *scyl2* was smaller as compared to WT [35]. In this study, by comparing the growth state of four different *Arabidopsis* lines (CK, *ZmSCYL2-OE*, *scyl2*, *scyl2* + *ZmSCYL2*), it was found that *ZmSCYL2-OE* grew best at the seedling stage, with larger leaves, longer root lengths, and the plants developed more rapidly. The mutants (*scyl2*) were relatively stunted in development, they had shorter root lengths, and the plants grew poorly. When the plants matured, the four *Arabidopsis* lines differed significantly in plant height, silique number, and silique length, with the *ZmSCYL2-OE* line having an absolute advantage in these aspects (Figure 5). It is evident that mutation and deletion of *SCYL2* affects both the vegetative and reproductive growth of the plants. After transferring *ZmSCYL2* into the mutant, the plants showed some recovery in growth status, with increases in plant height, number of siliques, and silique length, basically reaching the level of the wild type. As in the case of *Arabidopsis*, the tobacco overexpression line (*ZmSCYL2-OE)* grew and developed faster, had greener leaves, longer root length, and greater plant growth relative to CK. *ZmSCYL2-OE* also had larger and fuller pods, and the fruiting period was 1–2 weeks earlier than the control (Figure 7). These results further verified that the expression of *ZmSCYL2* results in phenotypic changes.

Species differences can lead to significant differences in the proportion of phytosterol composition. Although the major phytosterols in both *Arabidopsis* and tobacco are campesterol, stigmasterol, and β-sitosterol, unlike *Arabidopsis*, where the major sterol is β-sitosterol, the most abundant sterol in tobacco is stigmasterol, which accounts for about 50–70% of the total sterols. When sterols were extracted from Arabidopsis seeds, the highest sterol content was found in the seeds of *ZmSCYL2-OE,* which was 1.33 times the CK and 2.17 times the *scyl2* (Figure 6A). The variation of sterol content in *Arabidopsis* seeds was consistent with the expression levels of *ZmSCYL2*. The higher the expression level of *ZmSCYL2*, the higher the accumulation of sterols and the better growth of plants; conversely, the lower the expression level of *ZmSCYL2*, the lower the accumulation of sterols and the worse the plant growth. In previous studies, *SCYL2* was suggested to be involved in vesicular transport, which is related to the accumulation of substances. This study also confirmed that the accumulation of sterols is closely related to the expression of *ZmSCYL2* through functional verification in *Arabidopsis*. The determination of phytosterols in tobacco leaves and seeds showed that the levels of sterols in both leaves and seeds of *ZmSCYL2-OE* were much higher than in the control (Figure 6B,C). Although the content of sterols in the *ZmSCYL2-OE* increased, the proportion of each component remained unchanged, indicating that *ZmSCYL2* had no selective specificity for the transport of various sterols. These results suggest that *ZmSCYL2* is closely related to plant growth; overexpression of *ZmSCYL2* not only promotes plant vegetative and reproductive growth but also facilitates sterol accumulation.

## 4. Materials and Methods

### 4.1. Plant Materials and Growth Conditions

The plant materials used in this study included maize, *Arabidopsis,* and tobacco. Seeds of 244 maize inbred lines provided by Prof. Jianbing Yan of Huazhong Agricultural University (Wuhan, China) were used to assess sterol content and GWAS. Of these lines, 190 were from tropical and subtropical regions, and 54 were from temperate regions. All maize plants were grown in a greenhouse at 25 °C under 14 h/10 h light/dark conditions.

*Arabidopsis thaliana* Columbia-0 (Col-0) was used in this study. Seeds were sterilized in 75% (*v*/*v*) ethanol and 0.1% (*v*/*v*) Triton X-100 and then planted on 1/2 MS sterile medium. After stratification at 4 °C for 3 days, the plates were transferred to a growth chamber at 22 °C with a 16 h/8 h light/dark cycle. After 10 days, when *Arabidopsis* plants had grown four leaves, they were transplanted from the plates to soil under the same growth conditions.

Tobacco (*Nicotiana tabacum* cv. Yun Yan 85) seeds were sterilized with 75% (*v*/*v*) ethanol for 1 min and 5% NaClO for 15 min, followed by washing three times with sterile water. The sterile seeds were germinated and grown on MS medium for 6 weeks and then used either for Agrobacterium tumefaciens-mediated transformation or transplantation into soil. Tobacco plants were grown in the growth chamber at 25 °C with a 16 h/8 h light/dark cycle.

### 4.2. Extraction and Quantification of Phytosterols

We collected embryos of maize, seeds of *Arabidopsis*, leaves, and seeds of tobacco for phytosterols extraction after desiccating them in an oven at 37 °C for 48 h. The extraction method of phytosterols was based on previous literature [38] with minor modifications. Briefly, 0.5 g tissues were grounded into powder in liquid nitrogen before being placed in a Soxhlet Apparatus, and then phytosterols were extracted with 20 mL of ethyl acetate at 80 °C for 6 h. After the solution cooled, 4 µg of 5α-cholestane (20 µL of 0.2 mg/mL in ethyl acetate) used as an internal standard was spiked into a 100 mL flask. The extracts were dried with a rotary evaporator (IKA^®^ RV 10, Stauffen, Germany) at 40 °C and saponified with 15 mL NaOH ethanol solution (0.5 mol/L) overnight. The saponification solution was extracted by ethyl acetate, NaOH aqueous solution (0.5 mol/L), and Na_2_SO_4_ aqueous solution (0.2 mol/L) several times and then dried. Then, 2 mL acetonitrile was added for resolution. Then, 200 µL of the solution was put into the chromatographic bottle, and after drying with nitrogen gas, added 50 µL pyridine and 80 µL *N*-methyl-*N*-(trimethylsilyl) trifluoroacetamide. The solution was dissolved overnight, and the volume was fixed to 500 µL with isooctane and then filtered for detection. Three biological replicates and three technical replicates were performed for each sample.

The quantitative analysis of phytosterols was performed by an Agilent 7890A-5975 GC-MS system (Santa Clara, CA, USA) equipped with an Agilent DB-5 MS column (30 m × 0.25 mm i.d., 0.25 μm) (Santa Clara, CA, USA). Helium was used as a carrier gas maintained at the pressure control flow of 62.1 kPa (1 mL/min flow rate). Samples (1 μL) were injected using a G4513A autosampler and a split/splitless injector with a split ratio of 10:1. The oven temperature was initially set at 80 °C and then increased at a rate of 20 °C/min to 300 °C for 30 min. The temperatures of the ion source and transfer line were set at 230 °C and 280 °C, respectively. The mass spectra were obtained by electron impact ionization at 70 eV in full scan mode, and the mass range was *m*/*z* 50–550. Phytosterols were identified based on the retention time and mass spectral data of the reference standards. The GC retention time of campesterol, stigmasterol, and β-sitosterol were 21.623, 22.246, and 23.456 min, respectively. Three biological replicates and three technical replicates were performed for each sample. The mean values of the replicates were used for further analysis.

### 4.3. GWAS Analysis

The 558,529 SNPs, population structure, genotypes, and kinship data were kindly provided by Prof. Jianbing Yan (http://www.maizego.org (accessed on 10 June 2021)) [39,40]. A panel consisting of 244 inbred lines was genotyped by MaizeSNP50 BeadChip with 558,529 SNPs. The quality of each SNP was checked; SNPs with poor quality (minor allele frequency < 0.05, missing percentage > 20%) were excluded from further analysis, while 440,827 high-quality SNPs remained for GWAS analysis. Phytosterol content was analyzed for GWAS using a mixed linear model (MLM) with TASSEL 5.0 software. Principal component (PC) analysis and linkage disequilibrium (LD) analysis were also performed by TASSEL 5.0 software. The LD decay distance of the whole genome was determined by PopLDdecay software (v3.40). Based on the average LD decay of 50 kb, significant SNPs within 50 kb were grouped into a QTL and the most significant SNP was selected as the dominant SNP. Significantly associated SNP whose physical distance was less than the LD decay distance was defined as a locus, and the 100 kb range (50 kb upstream and 50 kb downstream of the SNP) was used to mine potential candidate genes.

### 4.4. RNA Extraction and Quantitative Real-Time PCR

In the qRT-PCR experiment, total RNA was extracted from plant tissues using an RNA extraction Kit (Vazyme, Nanjing, China). Genomic DNA was degraded with RNase-Free DNaseI (TaKaRa, Tokyo, Japan) by incubation in the supplied buffer for 20 min at room temperature. The purity and integrity of the RNA were determined by agarose gel electrophoresis and the A260/A230 and A260/A280 ratios. To generate first-strand cDNA, 1 μg of total RNA was reverse transcribed using a Clontech kit (TaKaRa, Tokyo, Japan) according to the manufacturer’s protocol. The cDNA was used as a template for quantitative real-time PCR (qRT-PCR) analysis. We measured the concentration of synthesized cDNA using a spectrophotometer or a specialized instrument to ensure equal amounts of cDNA were used in subsequent qRT-PCR reactions. qRT-PCR was performed using FastStart Universal SYBR Green Master (Roche, Shanghai, China) and StepOnePlus Real-Time PCR System (Applied Biosystems, Foster, CA, USA). The primers are listed in Supplementary Table S2. qRT-PCR reactions were performed using the SYBR^®^ Premix Ex TaqTM kit (TaKaRa, Tokyo, Japan) with the following conditions: 95 °C for 10 min, then 40 cycles of 95 °C for 15 s and 60 °C for 30 s. Relative gene expression levels were calculated by the 2^−∆∆Ct^ method [41]. Three independent experiments were performed for all reactions.

### 4.5. Cloning of ZmSCYL2 and Bioinformatics Analysis

The sequence of *ZmSCYL2* (GenBank accession number GRMZM2G061681) was obtained through the maize genome database (http://www.maizesequence.org/index.html (accessed on 8 September 2021)). Embryos of maize B73 inbred lines were used for RNA extraction. The specific method is as above in Section 4.4. For cloning of *ZmSCYL2*, the full-length cDNA sequence was amplified by PCR. Primers were: *ZmSCYL2*-F (5′-CCGCTCGAGATGGCGCTCAACATGAAGACC-3′) and *ZmSCYL2*-R (5′-TGCTCTAGACTAAAGTAAATCCAGGATAGGTTGTTGTC-3′).

The conserved domains of ZmSCYL2 protein sequences were predicted through the conserved domain database on the NCBI website (https://www.ncbi.nlm.nih.gov/Structure/cdd (accessed on 21 September 2021)). Multiple sequence alignment was performed using the ClustalW program. Phylogenetic analysis was conducted by using MEGA5.1 adopted with Poisson correction distance and presented using a circular tree view. Tree support was assessed using the bootstrap method with 1000 replicates.

### 4.6. Subcellular Localization of ZmSCYL2-GFP Fusion Protein

To investigate the subcellular localization of ZmSCYL2 in maize, the coding sequence of *ZmSCYL2,* excluding the termination codon, was amplified by PCR, where the SpeI and XbaI sites were underlined. The PCR products were digested with SpeI and XbaI and were cloned in the GFP gene in binary vector pCAMBIA1305 to produce the ZmSCYL2-GFP fusion protein under the control of the CaMV 35S promoter. The primers used for the construction were: *SCYL2*-GF (5′-ACTAGTATGGCGCTCAACATGAAGACC-3′) and *SCYL2*-GR (5′-TCTAGAAAGTAAATCCAGGATAGGTTGTTGTCCT-3′). Transient expression of the ZmSCYL2-GFP fusion protein in maize protoplast was conducted according to the method described previously [42]. RFP: MAN49 was used as a marker for Golgi. GFP and RFP signals were captured by an Olympus Confocal Laser Scanning Microscope (Olympus, Tokyo, Japan).

### 4.7. Generation of Transgenic Plants for Arabidopsis and Tobacco

The full-length cDNA sequence (2880 bp) of *ZmSCYL2* was inserted into the pCAMBIA2300 vector at the KpnI and XbaI sites under the control of the CaMV35S promoter. The constructed plasmid was transferred to *Agrobacterium tumefaciens* GV3101; the strains were used to transform *Arabidopsis* wild-type (Col-0) and mutant plants by the floral dip method [43]. T1 transgenic lines were harvested and screened on 1/2 MS mediums with 50 mg/L kanamycin (TaKaRa, Tokyo, Japan). The surviving seedlings were planted into the soil and identified by PCR. T2 seeds were sterilized and then planted on 1/2 MS mediums with 50 mg/L kanamycin. After stratification at 4 °C for 3 days, the plates were transferred to the growth chamber. One week later, plants were transplanted from plates into soil and maintained in the growth chamber at 22 °C with a 16 h/8 h light/dark cycle. T3 transgenic plants were used for further analysis.

The *Agrobacterium* strain used for tobacco transformation is the same as the *Arabidopsis* transformation. Tobacco seeds were sterilized and then planted on MS mediums. The plants were transformed by the leaf disk method [44], and transgenic shoot buds were directly generated from leaf disks. The T0 transgenic plants were screened out for propagation by PCR. Primers used for verification were: *SCYL2*-TF (5′-CAATCCCACTATCCTTCGCAAGACCC-3′) and *SCYL2*-TR (5′-TGCTCTAGACTAAAGTAAATCCAGGATAGGTTGTTGTC-3′). T3 transgenic plants were used for further analysis.

### 4.8. Identification and Complementation of Arabidopsis Mutants

*Arabidopsis* mutants (Sail-382-D06.v1, T-DNA insertion) in the Columbia background were obtained from the *Arabidopsis* Biological Resource Center. Homozygous lines were identified by PCR using the following specific primers: *ATB1*-LP (5′-GGTAAAGTGAACCTGCTGTCG-3′), *ATB1*-RP (5′-AATTGGATTCAAGTCGGAAGG-3′), and BP-SALK (5′-ATTTTGCCGATTTCGGAAC-3′). The floral dip method was conducted on homozygous mutants to generate transgenic complementation plants. Transgenic plants were selected using PCR analysis.

## 5. Conclusions

In the present study, phytosterol contents were measured in a panel of 244 maize inbred line embryos. GWAS were used to predict the possible candidate genes responsible for phytosterol accumulation. *ZmSCYL2* was tentatively identified to be associated with phytosterol content, followed by transgenic studies; significant enhancement of phytosterol levels was observed in transgenic *Arabidopsis* and tobacco, revealing that *ZmSCYL2* played an important role in plant growth and accumulation of phytosterols. Identification and characterization of phytosterol-related genes in maize will not only help us gain insight into the molecular mechanisms regulating phytosterol content in maize and other important cereal crops but can also be used for germplasm improvement through marker-assisted selection in breeding to improve sterol content and enhance the nutritional value of maize.

## Data Availability

All data are displayed in the manuscript and Supplementary Materials.

## Supplementary Materials

The following supporting information can be downloaded at: https://www.mdpi.com/article/10.3390/ijms241210411/s1.
