# Supplementary material for: Identification and Functional Characterization of ZmSCYL2 Involved in Phytosterol Accumulation in Plants
Source: Int J Mol Sci. 2023 Jun 20;24(12):10411. doi: 10.3390/ijms241210411 (PMC10299633; doi:10.3390/ijms241210411)
Supplement: Supplementary file 1 [file ijms-24-10411-s001.zip › ijms-2410971-supplementary.pdf]

Table S1. Statistic of phytosterol contents in 244 maize inbred lines

No.	Name	Campesterol		Stigmasterol		β-Sitosterol	
		Content (mg/g)	SD	Content (mg/g)	SD	Content (mg/g)	SD
1.	384-2	1.814	0.009	0.259	0.002	11.649	0.024
2	CML326	1.227	0.005	0.227	0.001	10.961	0.017
3	Ji63	2.888	0.012	0.207	0.002	9.007	0.078
4	CIMBL131	1.199	0.028	3.107	0.010	7.758	0.127
5	IRF314	1.870	0.002	0.520	0.001	9.164	0.010
6	CIMBL45	2.314	0.003	0.524	0.001	8.660	0.019
7	CIMBL99	1.691	0.014	0.258	0.006	9.395	0.047
8	CIMBL104	2.781	0.005	0.384	0.001	7.861	0.014
9	CML307	1.275	0.003	0.801	0.001	8.878	0.019
10	By804	2.239	0.006	0.304	0.003	8.204	0.024
11	CIMBL58	1.619	0.014	0.470	0.004	8.323	0.045
12	CIMBL48	1.887	0.003	0.475	0.001	7.990	0.032
13	CIMBL96	2.634	0.007	0.537	0.003	7.023	0.048
14	CIMBL124	2.006	0.005	0.401	0.002	7.787	0.016
15	04K5686	2.007	0.001	0.431	0.002	7.435	0.005
16	CIMBL120	2.228	0.004	0.641	0.002	6.929	0.033
17	5213	1.437	0.006	0.429	0.002	7.872	0.020
18	CIMBL74	2.756	0.005	0.285	0.002	6.611	0.030
19	CIMBL154	1.768	0.001	0.547	0.006	7.237	0.009
20	Yan414	1.383	0.005	0.408	0.003	7.756	0.038
21	CIMBL86	1.816	0.001	0.452	0.002	7.028	0.000
22	9642	1.796	0.005	0.331	0.003	6.975	0.022
23	MO113	0.890	0.001	0.270	0.001	7.915	0.014
24	CML20	1.683	0.001	0.639	0.002	6.581	0.015
25	CIMBL17	1.244	0.008	0.164	0.002	7.480	0.022
26	CML223	1.700	0.003	0.500	0.001	6.606	0.006
27	CIMBL156	1.946	0.004	0.287	0.001	6.511	0.020
28	Ji853	2.107	0.002	0.205	0.001	6.425	0.015
29	BT1	1.003	0.016	0.161	0.004	7.562	0.049
30	Dan3130	0.715	0.001	0.266	0.001	7.675	0.008

31	5237	1.756	0.008	0.508	0.004	6.348	0.023
32	CIMBL43	1.395	0.000	0.728	0.000	6.456	0.007
33	CIMBL49	2.239	0.001	0.399	0.001	5.897	0.008
34	CML32	1.871	0.016	0.544	0.005	5.991	0.076
35	By4839	1.375	0.003	0.383	0.001	6.579	0.010
36	TT16	1.299	0.006	0.455	0.001	6.521	0.024
37	CIMBL6	1.448	0.006	0.525	0.001	6.228	0.021
38	By815	2.142	0.005	0.362	0.001	5.674	0.040
39	CML470	1.246	0.002	0.427	0.000	6.439	0.012
40	Zheng29	1.474	0.004	0.190	0.002	6.319	0.019
41	CML327	1.628	0.004	0.282	0.002	5.993	0.016
42	CIMBL122	1.648	0.003	0.335	0.002	5.905	0.013
43	IRF291	1.266	0.003	0.365	0.001	6.216	0.011
44	Shen137	1.220	0.009	0.444	0.004	6.159	0.034
45	B111	1.547	0.005	0.424	0.003	5.837	0.016
46	CML432	1.276	0.006	0.730	0.002	5.801	0.028
47	M153	0.920	0.012	0.286	0.003	7.125	0.037
48	CML364	1.483	0.003	0.231	0.002	5.991	0.014
49	GEMS16	1.362	0.002	0.393	0.001	5.931	0.005
50	CML166	1.401	0.012	0.350	0.008	5.777	0.078
51	CIMBL133	0.747	0.005	0.214	0.002	6.492	0.017
52	CIMBL65	1.089	0.004	0.742	0.001	5.622	0.006
53	Zheng30	1.381	0.014	0.165	0.004	5.886	0.100
54	CML473	1.195	0.003	0.450	0.000	5.781	0.006
55	150	0.992	0.002	0.265	0.001	6.110	0.003
56	CIMBL90	1.525	0.005	0.355	0.003	5.474	0.007
57	CML285	0.792	0.002	0.154	0.002	6.370	0.006
58	CML192	1.107	0.003	0.518	0.001	5.644	0.007
59	TY8	1.451	0.001	0.326	0.002	5.447	0.004
60	CIMBL121	0.889	0.004	0.719	0.000	5.607	0.014
61	1462	1.034	0.014	0.282	0.003	5.889	0.053
62	CML465	0.841	0.003	0.197	0.001	6.113	0.005
63	CML170	1.133	0.004	0.399	0.001	5.579	0.012
64	R08	1.162	0.005	0.262	0.001	5.678	0.013

65	CML479	1.293	0.002	0.354	0.002	5.438	0.032
66	CIMBL125	1.216	0.003	0.288	0.001	5.544	0.011
67	CML172	1.401	0.002	0.274	0.001	5.295	0.009
68	GY386B	0.950	0.001	0.093	0.003	5.904	0.004
69	CIMBL52	1.398	0.002	0.458	0.001	5.064	0.010
70	CIMBL51	1.325	0.004	0.543	0.002	5.018	0.018
71	CML471	1.120	0.001	0.291	0.000	5.463	0.003
72	Sy1077	1.103	0.006	0.428	0.001	5.296	0.028
73	Qi205	1.279	0.003	0.315	0.001	5.145	0.018
74	CML433	1.061	0.003	0.400	0.001	5.270	0.016
75	LY042	0.751	0.002	0.537	0.001	5.405	0.009
76	CIMBL34	1.441	0.003	0.503	0.002	4.700	0.014
77	CIMBL18	0.782	0.028	0.183	0.003	5.593	0.010
78	Dong46	1.772	0.006	0.399	0.003	4.286	0.025
79	CIMBL32	1.451	0.005	0.300	0.001	4.655	0.032
80	CIMBL33	1.193	0.003	0.318	0.001	4.836	0.011
81	CML118	1.714	0.006	0.273	0.000	4.288	0.021
82	CIMBL102	0.936	0.010	0.333	0.002	4.996	0.037
83	W138	1.599	0.004	0.432	0.002	4.234	0.015
84	Lv28	1.142	0.002	0.546	0.001	4.569	0.004
85	CIMBL63	1.248	0.002	0.434	0.001	4.538	0.012
86	Dan4245	0.711	0.003	0.213	0.001	5.247	0.010
87	835a	0.777	0.003	0.206	0.001	5.132	0.003
88	CIMBL82	1.361	0.002	0.365	0.000	4.367	0.008
89	Lx9801	1.252	0.008	0.420	0.002	4.341	0.039
90	CIMBL56	0.803	0.002	0.227	0.000	4.962	0.007
91	CML29	1.253	0.003	0.415	0.105	4.308	0.017
92	CIMBL30	1.283	0.008	0.353	0.003	4.320	0.034
93	TY5	1.275	0.008	0.252	0.003	4.429	0.025
94	CIMBL146	0.620	0.000	0.377	0.001	4.923	0.009
95	CML27	0.988	0.003	0.425	0.002	4.473	0.013
96	CIMBL53	1.035	0.003	0.742	0.001	4.077	0.025
97	P178	1.044	0.005	0.303	0.000	4.501	0.005
98	TY7	1.239	0.005	0.242	0.005	4.356	0.024

99	CIMBL107	1.074	0.006	0.389	0.001	4.359	0.028
100	DE.EX	0.957	0.008	0.236	0.001	4.623	0.017
101	CIMBL150	1.126	0.003	0.271	0.001	4.281	0.009
102	CIMBL23	0.767	0.009	0.222	0.003	4.682	0.022
103	CML287	0.745	0.003	0.218	0.003	4.706	0.011
104	CIMBL83	0.908	0.009	0.164	0.003	4.559	0.025
105	CML324	0.800	0.004	0.260	0.002	4.540	0.038
106	647	1.084	0.005	0.281	0.000	4.215	0.017
107	Zheng653	1.293	0.002	0.367	0.000	3.908	0.011
108	GEMS37	1.059	0.001	0.397	0.001	4.080	0.015
109	CML361	0.920	0.002	0.205	0.001	4.384	0.013
110	Dan599	0.741	0.003	0.142	0.001	4.547	0.007
111	9782	0.659	0.003	0.419	0.001	4.298	0.017
112	CIMBL47	1.032	0.001	0.171	0.001	4.085	0.011
113	CML226	0.875	0.011	0.359	0.005	4.037	0.033
114	CML290	0.436	0.003	0.254	0.002	4.581	0.007
115	CIMBL153	1.174	0.002	0.337	0.002	3.751	0.010
116	CIMBL129	0.599	0.004	0.125	0.004	4.513	0.023
117	LXN	0.483	0.004	0.110	0.002	4.493	0.012
118	CIMBL100	0.999	0.003	0.466	0.002	3.598	0.014
119	CIMBL16	0.929	0.004	0.345	0.002	3.754	0.012
120	HTH-17	0.771	0.002	0.650	0.001	3.602	0.007
121	CIMBL138	0.672	0.002	0.807	0.002	3.535	0.011
122	975-12	0.768	0.001	0.265	0.000	3.823	0.006
123	GEMS2	0.807	0.001	0.188	0.002	3.855	0.006
124	CML423	0.941	0.009	0.285	0.002	3.587	0.034
125	Ry697	0.897	0.001	0.213	0.001	3.696	0.006
126	CIMBL78	0.701	0.004	0.161	0.002	3.906	0.014
127	Liao138	0.837	0.007	0.331	0.003	3.923	0.026
128	CML422	0.872	0.001	0.133	0.008	3.691	0.004
129	CIMBL136	1.127	0.002	0.267	0.001	3.278	0.006
130	CIMBL73	0.523	0.003	0.201	0.006	3.905	0.032
131	CIMBL95	0.875	0.005	0.231	0.006	3.447	0.025
132	832	0.873	0.006	0.269	0.003	3.406	0.050

133	CML298	0.852	0.004	0.159	0.002	3.535	0.015
134	CIMBL88	0.942	0.010	0.135	0.003	3.465	0.045
135	CIMBL9	0.727	0.003	0.400	0.002	3.358	0.016
136	CY72	0.867	0.010	0.296	0.002	3.277	0.048
137	04K5702	0.972	0.008	0.304	0.004	2.836	0.028
138	CIMBL93	0.593	0.005	0.253	0.001	3.231	0.010
139	CIMBL13	0.817	0.003	0.264	0.001	2.905	0.009
140	CIMBL91	0.567	0.002	0.163	0.000	3.060	0.006
141	CML139	0.539	0.001	0.300	0.001	2.931	0.003
142	CIMBL142	0.726	0.001	0.124	0.001	2.908	0.001
143	CML468	0.469	0.001	0.183	0.001	2.962	0.006
144	CML411	0.400	0.010	0.273	0.002	2.888	0.059
145	Gy237	0.450	0.002	0.147	0.001	2.956	0.005
146	B11	0.366	0.000	0.282	0.001	2.898	0.000
147	CML191	0.753	0.002	0.211	0.002	2.567	0.015
148	CIMBL76	0.773	0.004	0.359	0.002	2.393	0.058
149	Zong31	0.581	0.004	0.171	0.001	2.769	0.013
150	CML360	0.377	0.002	0.231	0.001	2.894	0.007
151	Liao159	0.602	0.003	0.107	0.002	2.573	0.012
152	CIMBL42	0.551	0.000	0.135	0.001	2.532	0.005
153	CIMBL11	0.447	0.003	0.170	0.001	2.414	0.019
154	CIMBL106	0.521	0.004	0.184	0.002	2.296	0.026
155	CIMBL89	0.174	0.005	0.128	0.002	2.646	0.018
156	CIMBL38	0.290	0.013	0.225	0.002	2.166	0.072
157	CML454	0.256	0.002	0.175	0.001	2.214	0.007
158	CIMBL75	0.360	0.001	0.159	0.002	2.092	0.012
159	CML51	0.195	0.002	0.152	0.001	2.216	0.013
160	CML426	0.377	0.001	0.077	0.002	2.029	0.010
161	CIMBL105	0.255	0.001	0.225	0.001	1.983	0.004
162	CML496	0.446	0.000	0.219	0.001	1.759	0.009
163	CIMBL128	0.187	0.004	0.051	0.001	2.176	0.012
164	SW1611	0.299	0.001	0.171	0.003	1.927	0.029
165	CML451	0.251	0.002	0.161	0.003	1.960	0.013
166	CIMBL22	0.195	0.003	0.091	0.001	2.070	0.020

167	CML134	0.373	0.004	0.213	0.003	1.707	0.018
168	CIMBL71	0.180	0.003	0.120	0.003	1.977	0.019
169	CML114	0.412	0.003	0.163	0.002	1.599	0.014
170	CML493	0.182	0.001	0.094	0.001	1.848	0.045
171	Nan21-3	0.263	0.004	0.092	0.003	1.769	0.026
172	CML115	0.322	0.000	0.165	0.001	1.602	0.009
173	CIMBL36	0.274	0.001	0.194	0.000	1.580	0.035
174	CIMBL14	0.345	0.015	0.084	0.002	1.609	0.058
175	Qi319	0.131	0.009	0.079	0.005	1.784	0.043
176	CIMBL143	0.268	0.004	0.080	0.002	1.633	0.024
177	CML40	0.368	0.002	0.167	0.002	1.441	0.030
178	CIMBL27	0.246	0.015	0.167	0.008	1.522	0.027
179	CML323	0.175	0.043	0.158	0.012	1.572	0.100
180	CML486	0.258	0.002	0.211	0.000	1.394	0.005
181	CML286	0.096	0.005	0.185	0.002	1.582	0.023
182	CIMBL67	0.167	0.005	0.093	0.003	1.596	0.039
183	CML325	0.217	0.010	0.133	0.004	1.486	0.048
184	CIMBL35	0.295	0.001	0.119	0.000	1.400	0.005
185	CIMBL110	0.289	0.001	0.094	0.005	1.426	0.007
186	CIMBL60	0.289	0.002	0.181	0.001	1.335	0.004
187	CIMBL54	0.231	0.007	0.123	0.007	1.426	0.033
188	CML31	0.215	0.002	0.129	0.002	1.409	0.008
189	CML26	0.340	0.002	0.251	0.002	1.148	0.019
190	CIMBL117	0.207	0.002	0.195	0.001	1.309	0.007
191	CML69	0.101	0.002	0.091	0.001	1.517	0.007
192	CIMBL40	0.217	0.002	0.176	0.002	1.249	0.035
193	CIMBL25	0.323	0.003	0.172	0.003	1.132	0.024
194	CML289	0.110	0.005	0.144	0.005	1.352	0.046
195	CIMBL87	0.428	0.002	0.060	0.002	1.095	0.007
196	CIMBL2	0.222	0.001	0.167	0.000	1.185	0.006
197	CIMBL139	0.260	0.002	0.170	0.001	1.143	0.013
198	CML415	0.293	0.004	0.133	0.002	1.137	0.046
199	CML116	0.133	0.002	0.090	0.003	1.307	0.011
200	CIMBL114	0.131	0.003	0.090	0.005	1.305	0.016

201	CIMBL145	0.128	0.002	0.097	0.002	1.290	0.007
202	CML171	0.279	0.005	0.053	0.000	1.172	0.017
203	CIMBL39	0.169	0.003	0.163	0.001	1.161	0.030
204	CIMBL85	0.195	0.003	0.321	0.003	0.922	0.007
205	CIMBL21	0.154	0.001	0.159	0.010	1.105	0.033
206	CIMBL57	0.152	0.002	0.142	0.006	1.110	0.026
207	CIMBL37	0.113	0.005	0.074	0.002	1.215	0.033
208	CIMBL29	0.168	0.001	0.116	0.001	1.118	0.012
209	CML304	0.116	0.003	0.079	0.004	1.200	0.023
210	CIMBL46	0.079	0.002	0.082	0.001	1.231	0.016
211	CIMBL1	0.206	0.003	0.144	0.002	1.033	0.008
212	CIMBL112	0.174	0.001	0.093	0.002	1.098	0.011
213	CIMBL72	0.152	0.003	0.135	0.001	1.071	0.017
214	CIMBL140	0.219	0.004	0.165	0.002	0.960	0.021
215	CML305	0.109	0.004	0.046	0.002	1.172	0.027
216	CIMBL20	0.222	0.002	0.182	0.002	0.889	0.022
217	CIMBL10	0.077	0.001	0.065	0.002	1.150	0.010
218	CIMBL24	0.177	0.002	0.123	0.000	0.986	0.016
219	CIMBL69	0.171	0.001	0.078	0.006	1.023	0.012
220	P138	0.133	0.005	0.054	0.001	1.055	0.018
221	CIMBL148	0.141	0.005	0.130	0.002	0.950	0.018
222	CIMBL119	0.124	0.002	0.125	0.001	0.935	0.010
223	CML28	0.137	0.002	0.132	0.003	0.907	0.012
224	CIMBL137	0.175	0.001	0.121	0.001	0.870	0.012
225	CIMBL155	0.107	0.003	0.083	0.001	0.972	0.010
226	CIMBL8	0.052	0.002	0.052	0.001	1.014	0.002
227	CIMBL132	0.147	0.002	0.125	0.001	0.845	0.013
228	CML168	0.169	0.001	0.144	0.000	0.797	0.014
229	CIMBL44	0.105	0.002	0.069	0.001	0.929	0.007
230	CML122	0.130	0.002	0.086	0.001	0.888	0.010
231	CIMBL126	0.195	0.003	0.144	0.001	0.740	0.015
232	CML50	0.108	0.001	0.094	0.002	0.839	0.004
233	CIMBL26	0.192	0.002	0.202	0.001	0.637	0.002
234	CIMBL157	0.126	0.003	0.115	0.001	0.783	0.002

235	CIMBL115	0.100	0.003	0.063	0.001	0.851	0.021
236	CIMBL7	0.096	0.001	0.049	0.002	0.787	0.015
237	CIMBL116	0.118	0.000	0.119	0.002	0.674	0.005
238	CIMBL31	0.126	0.001	0.098	0.001	0.671	0.013
239	CIMBL101	0.165	0.001	0.087	0.000	0.637	0.003
240	CIMBL144	0.082	0.001	0.083	0.002	0.715	0.003
241	CML121	0.086	0.001	0.109	0.001	0.682	0.002
242	CIMBL5	0.115	0.001	0.076	0.003	0.656	0.010
243	CIMBL92	0.104	0.003	0.079	0.001	0.656	0.011
244	CML130	0.071	0.001	0.077	0.001	0.548	0.010

Table S2. The primer sequences for qRT-PCR

**No.**	Name	Primer sequences
1.	*ZmSCYL2*-F	5'-AAACAGGCTGTGCTTCCTCGTGT-3'
2	*ZmSCYL2*-R	5'-GCCGAACCGTCTCCAAGATACCC-3'
3	*ZmActin*-F	5'-GGGATTGCCGATCGTATGAG-3'
4	*ZmActin*-R	5'-GAGCCACCGATCCAGACACT-3'
5	*AtActin*-F	5'-GGTGTCATGGTTGGTATGGGTC-3'
6	*AtActin*-R	5'-CCTCTGTGAGTAGAACTGGGTGC-3'
7	*NtL25*-F	5'-CCCCTCACCACAGAGTCTGC-3'
8	*NtL25*-R	5'-AAGGGTGTTGTTGTCCTCAATCTT-3'

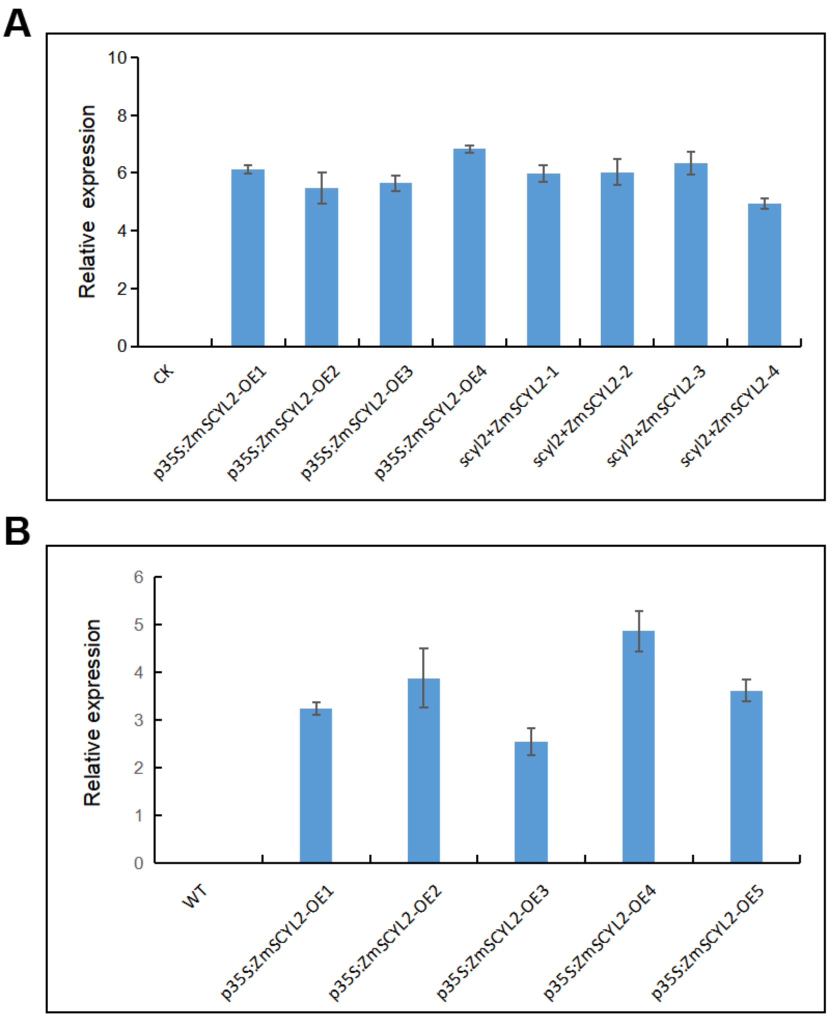

Figure S1. Relative expression levels of *ZmSCYL2* in transgenic plants. (A) Comparison the relative expression levels of *ZmSCYL2* in CK, *ZmSCYL2*-OE and *scyl2*+*ZmSCYL2* of *Arabidopsis*. (B) Comparison the relative expression levels of *ZmSCYL2* in WT and *ZmSCYL2*-OE of tobacco.
